# HTLV-1 viral oncogene HBZ induces osteolytic bone disease in transgenic mice

**DOI:** 10.18632/oncotarget.20565

**Published:** 2017-08-27

**Authors:** Alison K. Esser, Daniel A. Rauch, Jingyu Xiang, John C. Harding, Nicole A. Kohart, Michael H. Ross, Xinming Su, Kevin Wu, Devra Huey, Yalin Xu, Kiran Vij, Patrick L. Green, Thomas J. Rosol, Stefan Niewiesk, Lee Ratner, Katherine N. Weilbaecher

**Affiliations:** ^1^ Department of Medicine, Division of Oncology, Washington University School of Medicine, St. Louis, MO, USA; ^2^ Department of Veterinary Biosciences, School of Veterinary Medicine, The Ohio State University, Columbus, OH, USA

**Keywords:** HTLV-1, ATL, leukemia, HBZ, bone

## Abstract

Adult T-cell leukemia/lymphoma (ATL) is an aggressive T cell malignancy that occurs in HTLV-1 infected patients. Most ATL patients develop osteolytic lesions and hypercalcemia of malignancy, causing severe skeletal related complications and reduced overall survival. The HTLV-1 virus encodes 2 viral oncogenes, Tax and HBZ. Tax, a transcriptional activator, is critical to ATL development, and has been implicated in pathologic osteolysis. HBZ, HTLV-1 basic leucine zipper transcription factor, promotes tumor cell proliferation and disrupts Wnt pathway modulators; however, its role in ATL induced osteolytic bone loss is unknown. To determine if HBZ is sufficient for the development of bone loss, we established a transgenic Granzyme B HBZ (Gzmb-HBZ) mouse model. Lymphoproliferative disease including tumors, enlarged spleens and/or abnormal white cell counts developed in two-thirds of Gzmb-HBZ mice at 18 months. HBZ positive cells were detected in tumors, spleen and bone marrow. Importantly, pathologic bone loss and hypercalcemia were present at 18 months. Bone-acting factors were present in serum and RANKL, PTHrP and DKK1, key mediators of hypercalcemia and bone loss, were upregulated in Gzmb-HBZ T cells. These data demonstrate that Gzmb-HBZ mice model ATL bone disease and express factors that are current therapeutic targets for metastatic and bone resident tumors.

## INTRODUCTION

Viral infections cause some of the most common malignancies worldwide including human papillomavirus (cervical cancer), hepatitis B and C viruses (liver cancer), and Epstein-Barr virus (lymphoma). The understanding of viral oncogene functions has uncovered important basic mechanisms of tumor biology that have been used to prevent and treat cancer. Human T cell Leukemia/Lymphoma Virus 1 (HTLV-1) is the only human retrovirus known to directly cause cancer [1]. Adult T cell leukemia/lymphoma (ATL) is an aggressive lymphoproliferative malignancy of T cells that develops in a subset of HTLV-1 infected patients usually after decades of clinical latency. The acute form is refractory to aggressive chemotherapy treatments and has a mean survival time of less than one year, demonstrating the need to better understand this disease [2-4].

The HTLV-1 genome contains 2 viral oncogenes, Tax and HBZ. Tax has oncogenic properties in mouse models of ATL [5, 6]. Tax is a transcription factor that acts as a co-activator for CREB binding proteins [7]. Enhanced NFAT-1, AP-1 and NF-κB pathway activation by Tax regulates lymphocyte proliferation and apoptosis [8, 9]. In addition, Tax facilitates virus transmission and increases genomic instability [10, 11]. Tax is highly immunogenic and expression is repressed in human ATL cells [12, 13]. While the oncogenic properties of Tax have been extensively investigated, HBZ was only identified as a viral oncogene within the last 15 years. HBZ is a helix-basic loop zipper protein transcribed from the antisense strand of the HTLV-1 genome. HBZ acts as a transcription factor to negatively regulate Tax and is the only viral protein expressed in both early and late stages of human ATL [14, 15]. HBZ regulates cellular proliferation, reduces cellular aging and inhibits senescence of infected cells through enhanced expression of hTERT and JunD and inhibition of p65 and p53 [16-19]. HBZ can also impair expression of DICER and some miRNAs through removal of JunD from the DICER promoter [20]. Recent reports demonstrate that HBZ has oncogenic and inflammatory properties in animal models [21, 22].

In addition to leukemia/lymphoma, many HTLV-1 associated ATL patients develop paraneoplastic hypercalcemia and osteolytic bone lesions, which commonly occur in metastatic solid tumors such as breast and prostate, but is much less common in hematologic malignancies [23, 24]. Inhibiting bone loss in patients with bone metastases and multiple myeloma, reduced bone fracture, bone pain and improved quality of life [25]. We have previously reported that Tax transgenic mice under the Granzyme B promoter model many aspects of human ATL and that osteoclast inhibitors could inhibit the hypercalcemia and osteolytic bone lesions in these mice [5, 26, 27]. However, Tax protein is infrequently detected in primary human ATL cells, even in patients with hypercalcemia and osteolytic bone lesions. We therefore determined if HBZ, which is constitutively expressed in most human ATL cells, affects osteoclasts and bone, independent of Tax. As we did for the Tax transgenic mice, we have generated mice expressing HBZ under the Granzyme B promoter (Gzmb-HBZ). ATL is a T cell malignancy and the Granzyme B promoter specifically targets HBZ expression to T cells and NK cells.

We found that Gzmb-HBZ mice developed spontaneous, palpable tumors, enlarged spleens and/or abnormal white blood cell counts at 18 months. Tumors were primarily composed of hematopoietic cells and included HBZ expressing cells, which were also present in the spleen and bone marrow. Gzmb-HBZ lymphoproliferative disease was transplantable into both Gzmb-HBZ and NSG mice. Importantly, Gzmb-HBZ mice had significant bone loss and hypercalcemia of malignancy, analogous to the pathologic bone destruction and hypercalcemia present in ATL patients. Inflammatory cytokines and cytokines that have been shown to effect bone (IL-6, IL-3, MCP-1) were increased in serum from Gzmb-HBZ mice. Splenic T cells had increased expression of factors known to promote bone loss, including RANKL, PTHrP and DKK1. Importantly, these factors are the target of therapies for bone metastases and multiple myeloma [28].

## RESULTS

### Gzmb-HBZ mice develop spontaneous tumors

**Figure 1:**
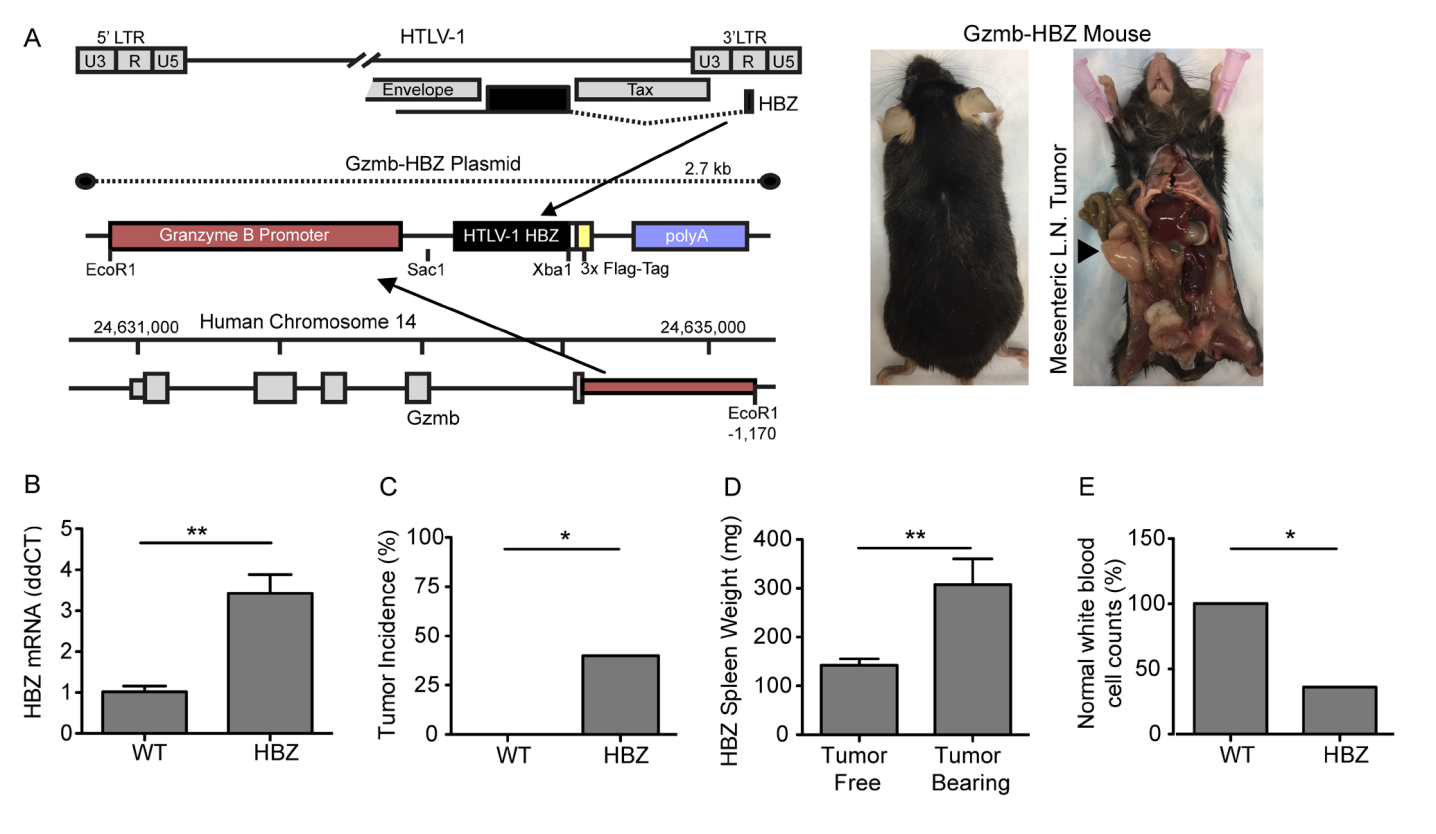
Granzyme B HBZ mice develop lymphoproliferative disease **A.** Granzyme B HBZ plasmid schematic and Gzmb-HBZ mouse. Arrow denotes a mesenchymal lymph node tumor in a Gzmb-HBZ mouse. **B.** HBZ mRNA expression in 4 month old WT and Gzmb-HBZ mice by Real-Time RT-PCR. **C.** Tumor incidence in 18-month WT and Gzmb-HBZ mice. **D.** Spleen weight in Gzmb-HBZ tumor free (*n* = 8) and tumor-bearing mice (*n* = 7). **E.** Percent of WT (*n* = 7) and Gzmb-HBZ (*n* = 15) mice with normal white blood cell counts (WBC). Normal WBC range was determined as the WT median +/- 2 standard deviations. **B.**-**D.** Statistical analysis represents mean +/- SEM. Statistical significance determined by non-parametric student’s *t*-test **B.**, **D.** or Fisher’s exact test **C.**, **E.** as appropriate. *P ≤ 0.05, **P ≤ 0.01, ***P ≤ 0.001.

HTLV-1 Tax transgenic mice develop leukemia and osteolytic disease; however, Tax is frequently repressed in late-stage HTLV-1 associated ATL when pathologic bone disease develops. The HTLV-1 viral gene, HBZ, is expressed at both early and late stages of human ATL and has been shown to cause expression of factors that can act on bone *in vitro* [29]. To evaluate the effect of HBZ on tumor development and bone loss *in vivo*, we generated transgenic mice expressing HBZ under the Granzyme B promoter (Gzmb-HBZ) (Figure 1A). To validate targeted HBZ expression, mRNA from Gzmb-HBZ and WT mouse T cells was measured. T cells were enriched using magnetic bead isolation from whole spleen and activated by culture on CD3/CD28 coated plates for 24 hours *ex vivo* to increase Granzyme B and HBZ expression. Gzmb-HBZ T cells expressed HBZ mRNA by qPCR analysis (Figure 1B). HBZ was not detected in T cells from WT mice.

To determine the oncogenic potential of HBZ in this model, 4-, 12- and 18-month cohorts of WT and Gzmb-HBZ mice were evaluated for tumor development, spleen size, complete blood counts (CBCs) and white cell differentials. There was no difference in these parameters between WT and Gzmb-HBZ mice at 4- and 12-months of age (Supplementary Figure 1A-1C). However, at 18 months, 40% of Gzmb-HBZ mice developed spontaneous tumors primarily localized to mesenteric and lymphoid tissues (Figure 1A, 1C). Spleen size was increased in Gzmb-HBZ tumor bearing mice (307 ± 52 mg, *n* = 7) compared to non-tumor bearing Gzmb-HBZ controls (142 ± 13 mg, *n* = 8) (Figure 1D). White blood cell (WBC) counts were assessed in Gzmb-HBZ and WT mice. The normal range was determined as the WT mean value +/- 2 standard deviations. 36% of Gzmb-HBZ mice had WBC counts outside of the normal range of 3.5 +/- 1.9 K/µl (Gzmb-HBZ WBC values of 0.8, 6.9, 8.4, 18.9 K/µl). All WT mice were within the normal range (Figure 1E). These data indicate that 66% of Gzmb-HBZ mice develop disease with overt tumors or lymphocyte abnormalities by 18 months.

### Gzmb-HBZ mice develop lymphoproliferative disease that is transplantable

**Figure 2:**
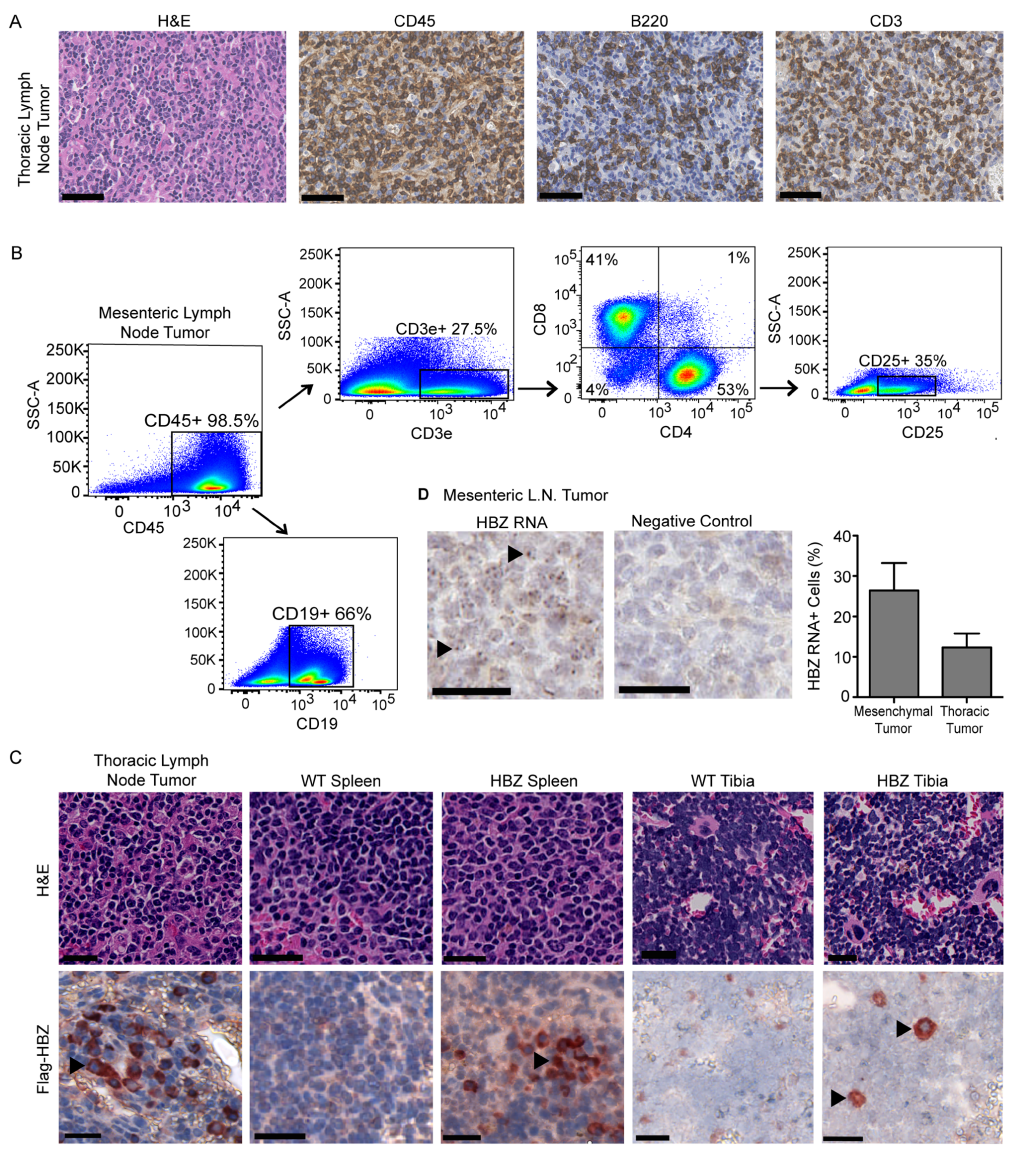
Lymphoid derived Gzmb-HBZ tumors express HBZ **A.** Gzmb-HBZ tumor immunohistochemistry (IHC) for hematopoietic (CD45), T cells (CD3e) and B/activated T cells (B220) in a thoracic lymph node-derived tumor. Scale bar = 50 µm. **B.** Flow cytometry analysis of T cell populations in a Gzmb-HBZ derived mesenteric lymph node tumor. **C.** Flag-HBZ expression in tumor, spleen and tibia of Gzmb-HBZ mice by IHC. Scale bar = 20 µm. **D.** HBZ RNA in Gzmb-HBZ tumor cells by *in situ* hybridization, representative images and quantification. Scale bar = 50 µm.

Tumors from Gzmb-HBZ mice were evaluated by immunohistochemistry (IHC) and flow cytometry. By IHC, Gzmb-HBZ tumors were composed of CD45+ hematopoietic cells including B220+ cells (B cells/activated T cells) and CD3e+ cells (T cells) (Figure 2A). Flow cytometry analysis of tumors from Gzmb-HBZ mice (*n* = 4) was performed to evaluate T cell subpopulations. Similar to IHC, 98.5% cells were hematopoietic (CD45+). Of the CD45+ cells, 27.5% were T cells (CD3e+) and 66% were B cells (CD19+) (Figure 2B). Of the CD3e+ T cell population, 41% were CD8+ T cells and 53% were CD4+ T cells. 35% of CD4+ cells were CD25+ T regulatory cells. These data demonstrate that a significant percent of cells in Gzmb-HBZ tumors were hematopoietic.

In this model, HBZ is flag-tagged and expressed in hematopoietic cells as a transgene regulated by the Granzyme B promoter. We therefore assessed HBZ expression in tumors and hematopoietic tissues including spleen and bone marrow by IHC. Expression of the flag epitope was detected in Gzmb-HBZ tumors using an anti-flag antibody (Figure 2C). Flag-HBZ positive cells were present in Gzmb-HBZ spleen and bone marrow of tibiae at 18 months. Flag-HBZ positive cells were not detected in the spleens of 4-month old mice (Supplementary Figure 1D). Two Gzmb-HBZ mice developed either a hepatic tumor or an epidermal tumor, both comprised of cells expressing cytokeratin (epithelial) but low levels of CD45. Flag-HBZ was not detected by IHC in these tumors. To confirm HBZ expression, HBZ RNA was detected by *in situ* hybridization. HBZ RNA was present in 12-25% of cells from Gzmb-HBZ derived tumors (Figure 2D and Supplementary Figure 1E). These data confirm that Gzmb-HBZ mice develop lymphoproliferative disease with localization of HBZ expressing cells to tumors and other hematopoietic tissues at 18 months.

**Figure 3:**
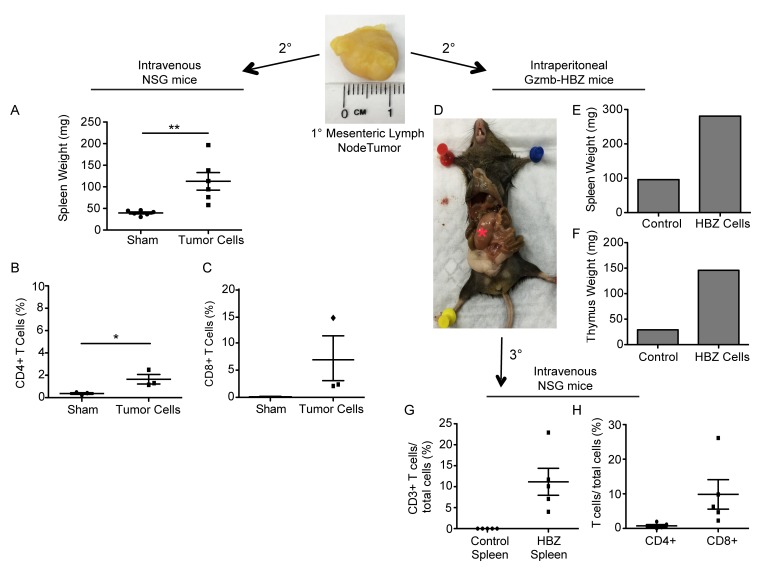
T cells from Gzmb-HBZ mice are transplantable **A.** Spleen weights from NSG mice implanted intravenously with PBS or mesenteric lymph node tumor cells (*n* = 5/group). **B.** Splenic CD4 and **C.** CD8 T cells from sham or tumor implanted mice. **D.** Image of a Gzmb-HBZ mouse 2-months post intraperitoneal tumor cell implantation with corresponding spleen weight **E.** and thymus weight **F.** *Red asterisk denotes a tumor. Percent of splenic CD3e T cells **G.** or CD4 and CD8 **H.** T cells from NSG mice serially implanted with tumor cells (D) or WT splenocytes (*n* = 5/group). All data reported as mean +/- SEM with statistical significance determined by Mann-Whitney U-test **A.** or unpaired *t*-test **B.** *P ≤ 0.05, **P ≤ 0.01, ***P ≤ 0.001.

A hallmark of malignant transformation is whether a transplanted tumor can proliferate in another host. Tumor cells from a primary Gzmb-HBZ mesenteric tumor (Figure 2B) or PBS were injected intravenously (iv) into NSG (*n* = 6) mice, which lack functional T cells, B cells, and NK cells, to look at proliferative and metastatic potential. Tumor development was assessed 2 months post tumor cell implantation. While overt tumors were not visible at 2 months, tumor cell implanted mice did develop enlarged spleens (110 ± 20 mg) compared to PBS injected controls (40 ± 2 mg) (Figure 3A). Flow cytometry analysis of splenocytes revealed a substantial difference in the percent of CD4+ and CD8+ T cells in HBZ tumor cell transplanted mice (1.6 ± 0.7, 6.3 ± 7.1, *n* = 3) *versus* PBS controls (0.36 ± 0.12, 0.12 ± 0.03, *n* = 3) (Figure 3B, 3C), indicating CD4 and CD8 T cells are transplantable.

We next evaluated whether Gzmb-HBZ tumor derived cells could be serially passaged in mice. Gzmb-HBZ cells derived from the primary mesenteric tumor (Figure 2B) were implanted into the peritoneal cavity of a Gzmb-HBZ mouse. At 2 months post injection, a palpable tumor was detected at the injection site (Figure 3D). The Gzmb-HBZ implanted mouse had increased spleen and thymus weights compared to the PBS injected control, supporting transplant of lymphoproliferative cells (Figure 3E, 3F). To distinguish implanted tumor cells from host cells, tumor cells from the secondary tumor were implanted into NSG mice (*n* = 5/group). Tumor cells or WT splenocytes were injected intravenously to determine metastatic potential. At 2 months post injection, overt metastases were not apparent. Splenocyte T cell populations were assessed by flow cytometry. CD3e+ T cells were detected in tumor cell implanted mice but were not detected in WT splenocyte implanted mice (Figure 3G). Tumor-transplanted mice had measurable CD4+ T cells and substantial CD8+ T cells present (Figure 3H). Together, these data demonstrate that cells derived from tumors in Gzmb-HBZ mice were transplantable.

### Decreased bone volume and hypercalcemia in Gzmb-HBZ mice

**Figure 4:**
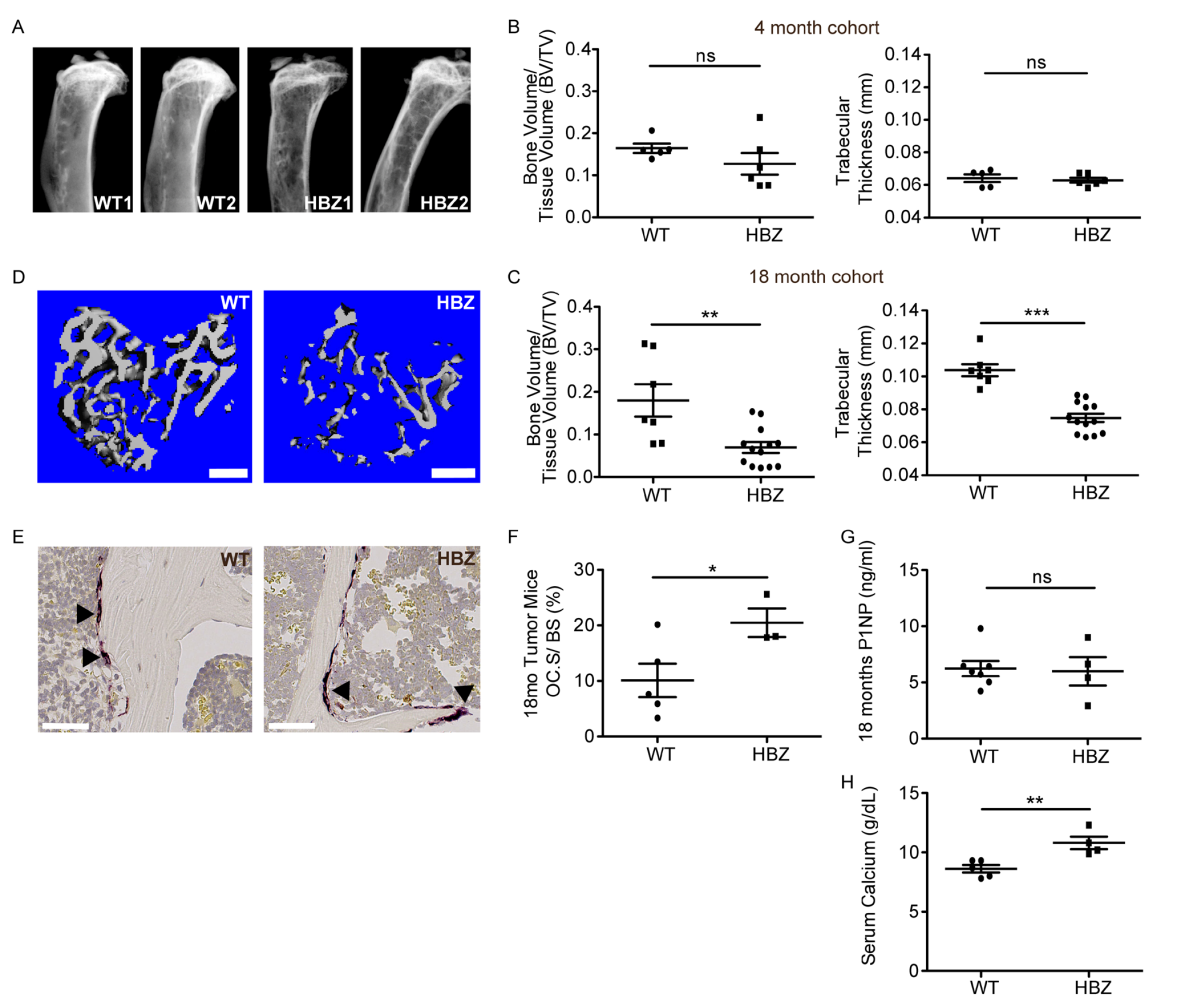
Gzmb-HBZ mice develop bone loss and hypercalcemia **A.** Representative X-ray images of WT and Gzmb-HBZ tibiae. MicroCT analysis of bone volume/tissue volume (BV/TV) and trabecular thickness (Tb.Th) at 4 months of age (*n* = 5/group) **B.** and 18 months of age **C.** in WT (*n* = 7) and Gzmb-HBZ (*n* = 13) mice. **D.** MicroCT representative images. Scale bar = 300 µm. TRAP+ cells (arrowheads) **E.** and quantification of osteoclast surface per bone surface **F.** in WT (*n* = 5) and Gzmb-HBZ (*n* = 3) tibiae. Scale bar = 50 µm. **G.** Enzyme-linked immunosorbent assay (ELISA) for osteoblast activity marker Procollagen Type I Intact N-terminal Propeptide (P1NP), measured in serum from Gzmb-HBZ (*n* = 4) and WT (*n* = 7) mice. **H.** Serum calcium levels in WT (*n* = 5) and Gzmb-HBZ (*n* = 4) mice at 18 months. All data reported as mean +/- SEM. Statistical significance was determined by unpaired *t*-test. *P ≤ 0.05, **P ≤ 0.01, ***P ≤ 0.001.

ATL is associated with bone loss and hypercalcemia of malignancy; therefore, bone loss was evaluated in the Gzmb-HBZ mice. Initial X-ray comparison of tibiae suggested Gzmb-HBZ had reduced bone compared to WT mice at 18 months (Figure 4A). At 4 and 12 months of age, 2 parameters of bone loss, bone volume/tissue volume (BV/TV) and trabecular thickness (Tb.Th.), were similar between WT and Gzmb-HBZ mice by microCT analysis (Figure 4B and Supplementary Figure 2A, 2B). By 18 months, BV/TV was significantly reduced in Gzmb-HBZ mice (-66%) compared to WT mice (0.06 ± 0.01, 0.18 ± 0.03). Tb.Th was also reduced in Gzmb-HBZ mice (-30%) compared to WT mice (0.07 ± 0.002 mm, 0.1 ± 0.003 mm) (Figure 4C, 4D). Osteoclasts (OC) are bone-resorbing cells frequently increased in number and function in ATL patients and patients with bone metastases. To determine if the OC number or OC surface/bone surface (OC.S/BS) was changed in Gzmb-HBZ mice, tartrate-resistant acid phosphatase (TRAP) stains of OC from histological sections of bone were evaluated. Osteoclast surface/bone surface (OC.S/BS) but not OC number (OC.N) was significantly increased in Gzmb-HBZ mice, suggesting enhanced bone resorption (Figure 4E, 4F and Supplementary Figure 2C). Some cancer cells modulate the bone microenvironment by altering the activity of bone forming osteoblasts. Procollagen Type I Intact N-terminal Propeptide (P1NP) is released during bone formation and can be used as a marker for osteoblast activity. WT and Gzmb-HBZ mice had similar levels of serum P1NP, indicating bone formation at 18 months is unchanged with HBZ expression (Figure 4G). Hypercalcemia due to resorption of bone is a serious complication for ATL patients. Serum calcium levels were significantly increased in Gzmb-HBZ mice (10.8 ± 0.53) compared to WT controls (8.62 ± 0.31) (Figure 4H). Thus, Gzmb-HBZ mice model the lymphoproliferative disease, bone loss and hypercalcemia present in HTLV-1 associated ATL.

### Gzmb-HBZ mice have increased inflammatory and bone-acting cytokines in serum

**Figure 5:**
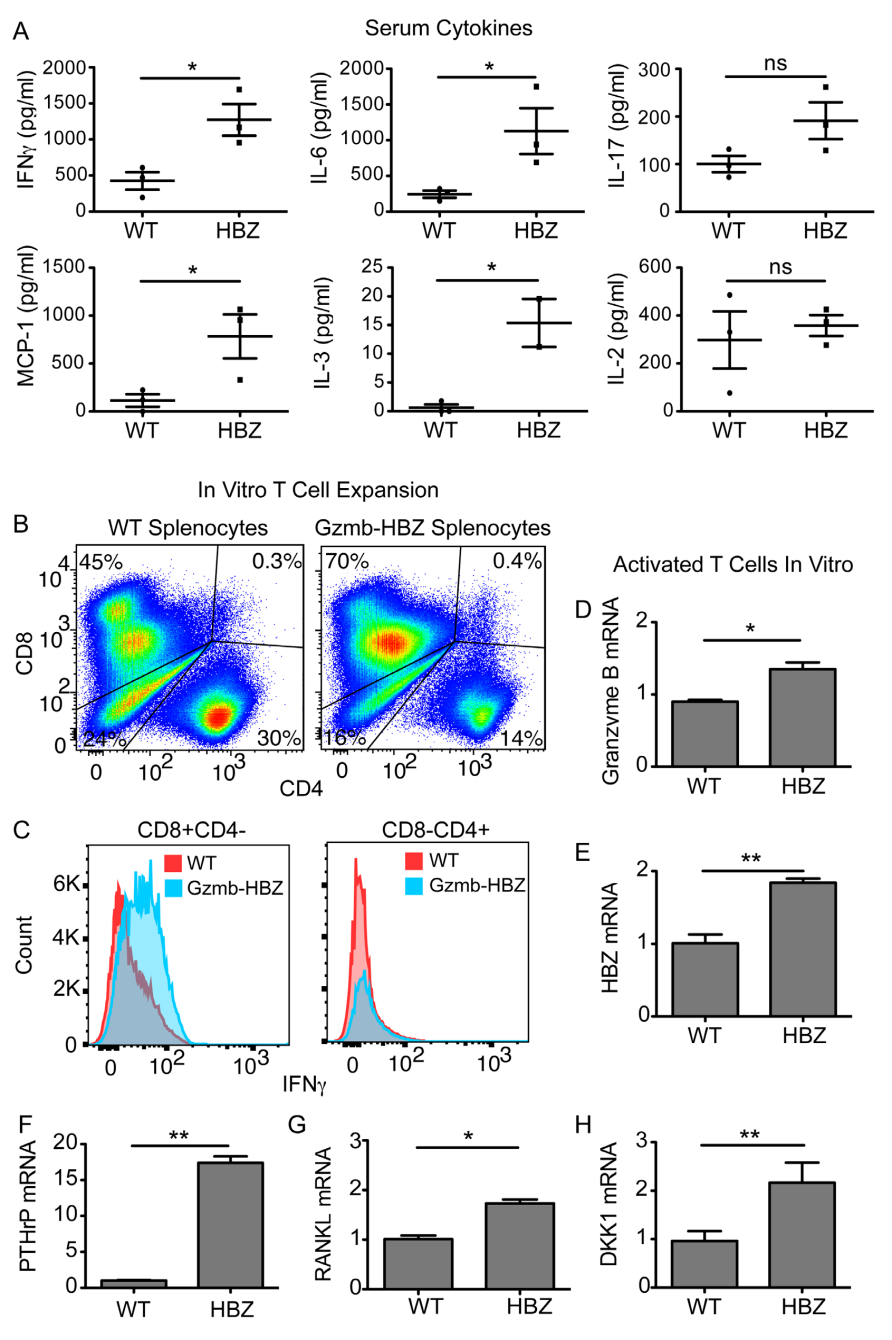
Gzmb-HBZ mice have systemic inflammation and increased T-cell derived bone-acting factors **A.** ELISA quantification of serum cytokines in 18-month WT (*n* = 3) and Gzmb-HBZ (*n* = 3) mice. Flow cytometry analysis of *ex vivo* expanded T cell populations **B.** and IFNγ expression **C.** from WT and Gzmb-HBZ splenocytes. Granzyme B **D.**, HBZ **E.**, PTHrP **F.**, RANKL **G.** and DKK1 **H.** mRNA expression in WT and Gzmb-HBZ *ex vivo* cultured, activated T cells. Data is representative of 2-3 biological replicates. All data reported as mean +/- SEM. Statistical significance determined by unpaired *t*-test. *P ≤ 0.05, **P ≤ 0.01, ***P ≤ 0.001.

Chronic inflammation and cancer can cause bone loss through multiple mechanisms. Tumors can secrete factors that act directly on bone resorbing osteoclasts (OC) or can have indirect effects on OCs through other cell types in the bone microenvironment. Serum from 18-month old, fasted Gzmb-HBZ (*n* = 3) and WT (*n* = 3) mice was analyzed for changes in cytokines and chemokines using a quantitative glass-slide antibody array. Inflammatory cytokines IFNγ and IL-6 were significantly increased in serum from Gzmb-HBZ mice compared to WT mice (Figure 5A). Furthermore, multiple factors that have been shown to directly and indirectly affect bone were significantly increased in Gzmb-HBZ mice compared to WT mice, including IL-6, MCP-1, and IL-3. Several factors trended toward increased levels in serum of Gzmb-HBZ mice such as IL-17, RANTES and IL-1β but did not reach statistical significance. The concentration of IL-2 in serum was similar between Gzmb-HBZ and WT mice, demonstrating that cytokines upregulated in Gzmb-HBZ transgenic mice were enriched in inflammatory and bone-acting factors.

To determine whether T-cells from HBZ mice express inflammatory factors, splenocytes from WT and Gzmb-HBZ mice were isolated and activated in culture on CD3/CD28 coated tissue culture plates followed by expansion in IL-2 containing media. Similar to CD8 T cell expansion in Gzmb-HBZ tumor cell transplanted mice, CD8 T cells were substantially increased in Gzmb-HBZ *ex vivo* T cell cultures (Figure 5B). Further, CD8 but not CD4 T cells had enhanced IFNγ by flow cytometry analysis (Figure 5C), demonstrating increased serum inflammatory factors in Gzmb-HBZ mice may in part be due to enhanced T cell expression.

Parathyroid hormone related protein (PTHrP) is an important mediator of hypercalcemia and osteolytic bone loss through upregulation of RANKL whereas; the Dickkopf-WNT signaling pathway inhibitor 1 (DKK1) inhibits osteoblast bone formation, thus altering the bone resorption/bone formation balance of the bone microenvironment towards osteolytic bone loss. To evaluate the effects of HBZ expression in T cells on expression of these bone modifying proteins, splenocytes from WT and Gzmb-HBZ mice were isolated and expanded in culture on CD3/CD28 coated tissue culture plates for 24hrs. Granzyme B and HBZ expression were increased in Gzmb-HBZ T cells compared to WT (Figure 5D, 5E). We then measured PTHrP, RANKL and DKK1 mRNA, which activate osteoclasts and inhibit osteoblast function to promote bone loss. PTHrP, RANKL and DKK1 mRNA were significantly increased in T cells from Gzmb-HBZ mice compared to WT controls (Figure 5F-5H). Thus, HBZ may enhance expression of bone-acting serum cytokines and other factors known to have osteolytic effects on bone and are targeted by current therapeutics.

## DISCUSSION

HTLV-1 induced ATL is associated with significant skeletal complications including hypercalcemia and osteolytic bone lesions. The HTLV-1 viral oncogene, Tax, regulates the expression of osteoclast-acting factors and may be a primary driver of bone pathology in ATL. We show for the first time, that HBZ transgenic mice also develop pathologic bone loss in addition to lymphoproliferative disease, suggesting that HBZ may also play a causative role in the pathogenesis of ATL induced bone loss. HBZ is a particularly compelling candidate for pathologic bone loss in ATL because Tax expression is frequently repressed in ATL cells, particularly at later stages, while HBZ is ubiquitously expressed in ATL cells and in all ATL disease stages [14].

We evaluated 4, 12 and 18-month cohorts of Granzyme B HBZ (Gzmb-HBZ) mice for tumor formation and bone loss. Tumors and hematopoietic changes were not evident until the 18-month time point. Interestingly, there is also a long latency between HTLV-1 infection and the development of ATL. We found that two-thirds of Gzmb-HBZ mice developed lymphoproliferative disease including palpable tumors, enlarged spleen and/or abnormal white blood cell counts. Overt tumors were present primarily in lymphoid and mesenteric tissues and were composed of significant numbers of CD45+ hematopoietic cells including CD3e+ T cells that encompass both CD4 and CD8 T cells.

We observed several key similarities between our Gzmb-HBZ mice and recently published CD4-HBZ transgenic mice [21]. The CD4-HBZ mice develop leukemia at older ages, around 16 months, with changes in hematopoietic profiles, splenic infiltration and systemic inflammation, as was observed in the Gzmb-HBZ mice. We also observed HBZ expression in CD4 T cells from Gzmb-HBZ mice. In contrast, enlarged lymph node tumors were characteristic of the Gzmb-HBZ mice, but were not reported in the CD4-HBZ mice. Further, CD4-HBZ mice developed dermatitis not present in the Gzmb-HBZ model. The different expression patterns between CD4 (CD4 T helper cells) and Granzyme B (mature cytotoxic T and NK cells) promoters may explain some of the phenotypic differences seen in these models.

Interestingly, independent expression of HTLV-1 oncogenes Tax and HBZ by the same promoter, Granzyme B, resulted in very different tumor phenotypes in transgenic mice. Although both developed tumors in lymphatic tissues, Gzmb-HBZ mice did not develop the peripheral subcutaneous tumors characteristic of Granzyme B Tax mice [5]. This may in part be attributed to the previously reported weaker oncogenic potential of HBZ compared to Tax. Indeed, HBZ mice develop lymphoproliferative disease after an 18-month latency as compared to the 6-9 month latency to malignancy in the Granzyme B Tax mice, further supporting that Tax is a more potent oncogene.

Characterization of malignant cells within tumors was more challenging than anticipated. NSG mice transplanted with cells from a primary Gzmb-HBZ tumor had detectable CD4 and CD8 T cells in the spleen. NSG mice implanted with serially passaged tumor cells, had measurable CD4 T cells present and substantial expansion of CD8+ T cells, suggesting lymphoproliferative disease was transplantable, a hallmark of malignant transformation. Importantly, Granzyme B is highly expressed in CD8 T cells and is expressed in some cytotoxic CD4 subsets. Recent studies have found that in HTLV-1 infected patients, infected CD8+ T cell undergo greater oligoclonal proliferation than infected CD4+ cells, however; CD8+ ATL is rare suggesting that clonal T-cell proliferation does not predispose patients to malignant disease [30]. In the CD4-HBZ transgenic mouse model, an ATL stem cell candidate (c-kit+/CD4-/CD8-) was identified and the c-kit-SCF signaling pathway was implicated in leukemia development. It would be interesting to determine if this population is present in the serially transplanted Gzmb-HBZ lymphoproliferative cell population [31].

Cells within Gzmb-HBZ tumors expressed HBZ protein and RNA. HBZ positive cells comprised a small number of total cells, making characterization of the malignant cells within tumors more difficult. It is possible that HBZ expression was lost in tumors, perhaps through promoter methylation [32]. Experiments using inducible systems could address the necessity of persistent HBZ expression and tumor formation. Another possibility is that low HBZ expression in the tumor could indicate that HBZ is indirectly effecting tumor formation through inflammation or other signaling pathways. Inflammation has been reported to increase tumor formation and loss of IL-15 promoted tumor development in the Granzyme B Tax model [33]. Importantly, the CD4-HBZ transgenic model also reported systemic inflammation that contributed to leukemia onset [21, 34]. We similarly found that the Gzmb-HBZ transgenic mice had inflammatory cytokines in the serum and in *ex vivo* T cell cultures. Thus, it is possible that indirect HBZ-mediated effects caused tumors in Gzmb-HBZ mice. Future studies will interrogate the role of HBZ and HBZ-associated factors in malignant transformation and tumor formation in transplantation studies. In addition, it has recently been reported that HBZ protein and RNA have distinct functions in T-cell proliferation and survival. HBZ protein has been shown to regulate transcription of immune-associated genes while RNA was associated with genes related to cell proliferation. It will be important to delineate the differential effects of RNA and protein on oncogenesis and bone loss in HTLV-1 associated ATL [15, 35].

In Gzmb-HBZ transgenic mice, development of bone loss and hypercalcemia coincided with lymphoproliferative disease, demonstrating HBZ-mediated bone loss *in vivo* for the first time. Bone is continuously remodeled and the process of bone resorption (osteoclasts) and bone formation (osteoblasts) is tightly coupled. Some diseases including cancer disrupt this coupling causing a net increase (prostate cancer) or decrease (breast cancer) of bone. Although osteoclast number was unchanged in Gzmb-HBZ and WT mice, osteoclast size was increased in Gzmb-HBZ mice. OC size is measured by length on the bone surface and a larger size can represent an increase in resorptive surface and accelerated bone loss. Osteoblast activity measured by serum collagen marker P1NP, was similar between Gzmb-HBZ and WT. It has been reported that by 18 months, there is little bone formation and this may explain why there was no difference in the osteoblast activity marker P1NP between WT and Gzmb-HBZ mice. Of note, Gzmb-HBZ tibiae did not have osteolytic lesions characteristic of a localized tumor effect but instead had an overall thinning of the trabeculae representative of systemic bone loss seen in some types of cancer, including breast cancer. We have previously shown that Tax transgenic mice (under the Granzyme B promoter) develop bone loss and hypercalcemia by 9 months of age. We now demonstrate that HBZ expression can have significant effects on bone loss, independent of Tax or other viral pathogenesis factors.

Since significant lymphoproliferative disease was not present in the bone marrow of Gzmb-HBZ mice and cells that express HBZ were detectable in bone marrow in only limited numbers, we assessed expression of bone-acting and pro-inflammatory factors in serum. Cytokines have important effects on bone resorptive osteoclasts and can induce systemic bone loss [25]. We found that factors known to promote bone loss in solid and hematologic cancers (IL-6, MCP-1 and IL-3) were significantly elevated serum from Gzmb-HBZ mice compared to WT mice, indicating HBZ-mediated changes in cytokine secretion may directly or indirectly accelerate bone loss in this model. In addition, chronic inflammation causes osteolytic bone loss in diseases such as rheumatoid arthritis. Gzmb-HBZ mice had elevated levels of serum inflammatory cytokines and inflammation could contribute to bone loss in this model.

Several factors including, IL-1, MIP1α, RANKL and PTHrP, have been implicated in ATL mediated hypercalcemia and bone loss. Parathyroid hormone-related protein (PTHrP) induces hypercalcemia and systemic bone loss, in part, through upregulation of RANKL, a critical factor in osteoclast formation, and enhanced bone resorption. PTHrP expression is increased in HTLV-1 infected cell lines, patient serum and in primary ATL cells, however little is known about the regulation of PTHrP expression by the HTLV-1 virus [23]. We found that in addition to Granzyme B and HBZ, PTHrP was significantly increased in T cells isolated from Gzmb-HBZ mice, providing the first *in vivo* evidence that HBZ could mediate hypercalcemia through enhanced PTHrP expression. Receptor activator of nuclear factor kappa-B ligand (RANKL) expression by some tumor types increases osteoclast formation and promotes bone resorption. Overexpression of the RANKL gene in ATL patients correlates with hypercalcemia [36]. In our transgenic mouse model, RANKL expression was significantly increased compared to WT mice, supporting a potential role for RANKL in HTVL-1 mediated bone loss. In addition, expression of the WNT pathway inhibitor Dickkopf-1 (DKK1) was increased by HBZ expression *in vitro* [29]. DKK1 inhibits bone-forming osteoblasts to promote bone loss in metastatic and hematopoietic malignancies [37]. We found that DKK1 expression was also increased in Gzmb-HBZ T cells compared to WT controls *in vivo*, suggesting HBZ may directly or indirectly effect DKK1 expression. Thus, in the Gzmb-HBZ mouse model, expression of PTHrP, RANKL and DKK1, known inducers of hypercalcemia and bone loss and therapeutic targets for osteoporosis and cancer, were increased in T cells.

In this work, we have shown that Gzmb-HBZ mice developed lymphoproliferative disease and model the bone loss and hypercalcemia present in ATL patients. We further identified elevated bone-acting factors in both serum and T cells from Gzmb-HBZ mice. Importantly, these factors have established roles in pathologic bone loss in osteoporosis, metastatic cancer and chronic inflammatory diseases. We thus demonstrated for the first time in an animal model, that the HTLV-1 oncogene HBZ might have an important role in ATL-mediated bone loss independent of Tax and suggest that HBZ-mediated bone loss may respond to current therapies for bone resident and metastatic tumors. Additional studies are needed to evaluate the effects of HBZ on bone loss in the context of the entire HTLV-1 genome.

## MATERIALS AND METHODS

### Animal studies

An independent transgenic Gzmb-HBZ mouse colony was derived and bred in the Weilbaecher lab from TAX-Luciferase-HBZ transgenic mice (C57Bl/6J; FVB), a generous gift from Dr. Lee Ratner. In the TAX-Luc-HBZ model (unpublished), the Granzyme B promoter regulates TAX and HBZ expression. The 5’LTR fragment of pHTE-1 regulates firefly luciferase (pGL-3; Promega). Individual Granzyme B TAX [5] and Granzyme B TAX-Luc [38] strains have been previously described. For evaluation, 4, 12 and 18-month cohorts of WT (*n* = 8), and Gzmb-HBZ mice (*n* = 8) were generated. A second cohort of 18-month Gzmb-HBZ mice was generated to facilitate evaluation of tumor heterogeneity (*n* = 7). Cohorts consisted of equal numbers of male and female mice. Animals were housed under pathogen-free conditions according to the guidelines of the Division of Comparative Medicine, Washington University (St. Louis, MO). Animal studies were performed under protocols approved and monitored by the Washington University Institutional Animal Care and Use Committee.

### Construction of the HBZ transgene

The Granzyme B promoter flanked by EcoRV sites (using DRGZBL and DRGZBR primers shown) was inserted into the SnaBI linearized pHBZ1 plasmid [39] . The purified 2.7kb transgene (produced using DRGZBL and DRGZBHBZR primers) was used for microinjections. Primers; DRGZBL: GGATATCGAATTCTATATTTTGAG, DRGZBR: ACGCGATATCCTGTTGTTTCCTCCTT and DRGZBHBZR: TTCCACAGCCAAGCTGGCCG.

### Quantitative RT-PCR

RNA was extracted from either cells or frozen tissue homogenized by mortar and pestle using the RNeasy Mini kit (Qiagen, Venlo, Netherlands) and cDNA generated with iScript (Bio-Rad, Hercules, CA, USA). Quantitative PCR was completed using SsoFast EVA Green Supermix (Bio-Rad). For HBZ expression, RNA was treated with DNase (Invitrogen) prior to RT-PCR. All steps were completed following the manufacturers instructions. Granzyme B (Forward-CCACTCTCGACCCTACATGG; Reverse-GGCCCCCAAAGTGACATTTATT), Cyclophilin A (Forward-AGCATACAGGTCCTGGCATC; Reverse- TTCACCTTCCCAAAGACCAC), PTHrP (Forward-CATCAGCTACTGCATGACAAGG; Reverse-GGTGGTTTTTGGTGTTGGGAG), DKK1 (Forward-TCCCAGAAGAACCACACTGA, Reverse-GTCTGATGATCGGAGGCAGA).

### Histology and immunohistochemistry

Tissues were fixed in 10% formalin for paraffin embedding. Tibias were decalcified in 14% EDTA prior to paraffin embedding. 5µm sections were stained by immunohistochemistry (IHC) with rat anti-mouse CD45 (BD Pharmingen #550539), CD3 (Dako #A0452), B220 (BD Pharmingen #550286). Flag-tagged HBZ expression was detected following citrate antigen retrieval with the anti-flag antibody (Sigma #F1804, 5ug/ml) and detected using the HistoMouse-Plus Broad Spectrum (AEC) kit (Life Technologies, catalog #849541). Tibias were stained with the tartrate-resistant acid phosphatase (TRAP) (Sigma-Aldrich) to visualize osteoclasts. The number and surface area of TRAP+ cells/ bone surface area were quantified at 5X magnification for WT (*n* = 7) and HBZ (*n* = 7) mice. Images were acquired using the NanoZoomer 2.0-HT System (Hamamatsu Photonics) or the Zeiss Axio Scan Z1.

### RNA *in situ* hybridization

Paraffin embedded tissue was stained for RNA *via in situ* hybridization (RNAscope Manual Assay 2.5HD - BROWN kit, ADC Biotechnology). Tissues were deparaffinized and pretreated with hydrogen peroxide, protease, and an antigen retrieval solution as directed. Antigen retrieval was performed in a pressure cooker for 30 minutes followed by incubation with the HBZ probe or the provided negative and positive controls. The HBZ probe (ADC Biotechnology) has a target region from 173-1363 in the HBZ RNA. The negative control probe hybridized the E. Coli gene *DapB* and the positive control probe hybridized the mouse housekeeping gene *Ppib*, Tissue sections were stained with DAB and hematoxylin, and mounted (Clearmount medium). Images were scanned using the Nanozoomer digital slide scanner (Hamamatsu Photonics). Five images at 60X magnification were taken randomly for each sample and positive cells/total cell number was quantified manually.

### Tumor implantation

Cells were isolated from a spontaneous mesenteric Gzmb-HBZ tumor by mechanical disruption through a 70um cell strainer and frozen in 7% DMSO containing media and stored in liquid nitrogen. NSG or Gzmb-HBZ mice were implanted by intravenous or intraperitoneal injection of 1x10^7^ tumor cells following twice PBS washes. In the Gzmb-HBZ mice, the secondary tumor was isolated mechanically as described and frozen and stored in liquid nitrogen. Thawed cells were washed twice in PBS and i.v. implanted in NSG mice at 1x10^7^ cells per mouse.

### Flow cytometry

Spleens were mechanically disrupted through a 70µm cell strainer. Cells were washed with PBS and red blood cells lysed (Sigma-Aldrich, #R7757). Cells were washed twice, centrifuged and blocked for 10 minutes on ice. Cells were resuspended in antibody diluent containing fluorophore-conjugated antibodies at 1:200 (ebioscience antibodies CD45, CD3e, CD4 and CD8). Cells were incubated 1 hour on ice, then washed twice in PBS and resuspended in FACS buffer (5% FBS in PBS) for analysis on the BD Bioscience LSRFortessa.

### MicroCT of WT and Gzmb-HBZ tibiae

Tibiae were scanned by micro-computed tomography (μCT) (μCT-40; Scanco Medical). The trabecular region from forty 2D slices (0.8 mm) was selected using contours inside the cortical shell on each 2D image, with the growth plate as a marker to determine a consistent location to start analysis. A 3D cubical voxel model of bone was built, and calculations were made for bone volume/tissue volume (BV/TV) and trabecular thickness (Tb.Th) and bone mineral density (BMD) calibrated against a hydroxyapatite phantom. A threshold of 300 (out of 1,000) was used to differentiate trabecular bone from non-bone.

### Mouse cytokine serum array

Serum was collected from WT (*n* = 3) and Gzmb-HBZ (*n* = 3) mice after 16 hours without food and was frozen at -80 degrees Celsius. Thawed serum was applied to a glass slide antibody array and processed according to manufacturer’s instructions (QAM-CYT-1-1, Raybiotech). Raybiotech completed slide scanning and data extraction. The primary author analyzed raw data. For analysis, background was subtracted and mean values were determined (3-4 replicates per cytokine) and converted from fluorescence unit to protein concentration by a standard curve best-fit regression specific for each cytokine.

### *Ex vivo* T cell culture

Spleens were gently mechanically disassociated through a 70um cell strainer. Isolated cells were plated in a tissue culture treated plate overnight at 37 degrees Celsius in media (RPMI, 5% FBS). Non-adherent cells were transferred to CD3e/CD28 coated 6-well plates in T cell media (RPMI, 5% FBS, 1% P/S, IL-2) for 24hrs at 37 degrees Celsius. Cells were collected, rinsed in PBS and RNA isolated by RNeasy RNA isolation kit (Qiagen). Remaining cells were plated in 6-well tissue culture plates with T cell media, expanded for 3 days and analyzed by flow cytometry as described above.

### Statistics

Data are shown as mean with error bars representing standard error of the mean (mean ± SEM), unless otherwise specified. Differences among experimental groups were analyzed using a two-tailed *t*-test or Fisher’s exact test (tumor incidence and WBC counts) as appropriate. Assumptions for *t*-test analyses (independent samples, approximately normal distributions) for samples *n* > 5 were sufficiently met, or satisfied if a random sample of *n* ≤ 5 were selected from an approximately normally distributed population. For data where the sample number was too small to determine normality (*n* ≤ 5 independent samples or biological replicates), but where the variance between groups was similar (using the F-test for equality of variances), two-tailed *t*-test analyses [40] were used where appropriate. For non-normally distributed data, differences among experimental groups were analyzed using a two-tailed Mann-Whitney U-test for unpaired samples. All tests were considered significant at *P* ≤ 0.05. Data analyses were complete using Prism 6 (GraphPad Software).

## SUPPLEMENTARY MATERIALS FIGURES



## Abbreviations

(HTLV-1): Human T cell leukemia/lymphoma virus
(ATL): Adult T cell leukemia/lymphoma
(Gzmb-HBZ): Granzyme B HBZ
(OC): Osteoclasts
(Tb.Th): Trabecular thickness
(BV/TV): Bone volume/ tissue volume
(OC.S/BS): Osteoclast surface/ bone surface
(TRAP): Tartrate-resistant acid phosphatase
