# Supplementary material for: HTLV-1 viral oncogene HBZ induces osteolytic bone disease in transgenic mice
Source: Oncotarget. 2017 Aug 27;8(41):69250–63. doi: 10.18632/oncotarget.20565 (PMC5642476; doi:10.18632/oncotarget.20565)
Supplement: Supplementary file 1 [file oncotarget-08-69250-s001.pdf]

HTLV-1 viral oncogene HBZ induces osteolytic bone disease in transgenic mice

SUPPLEMENTARY MATERIALS

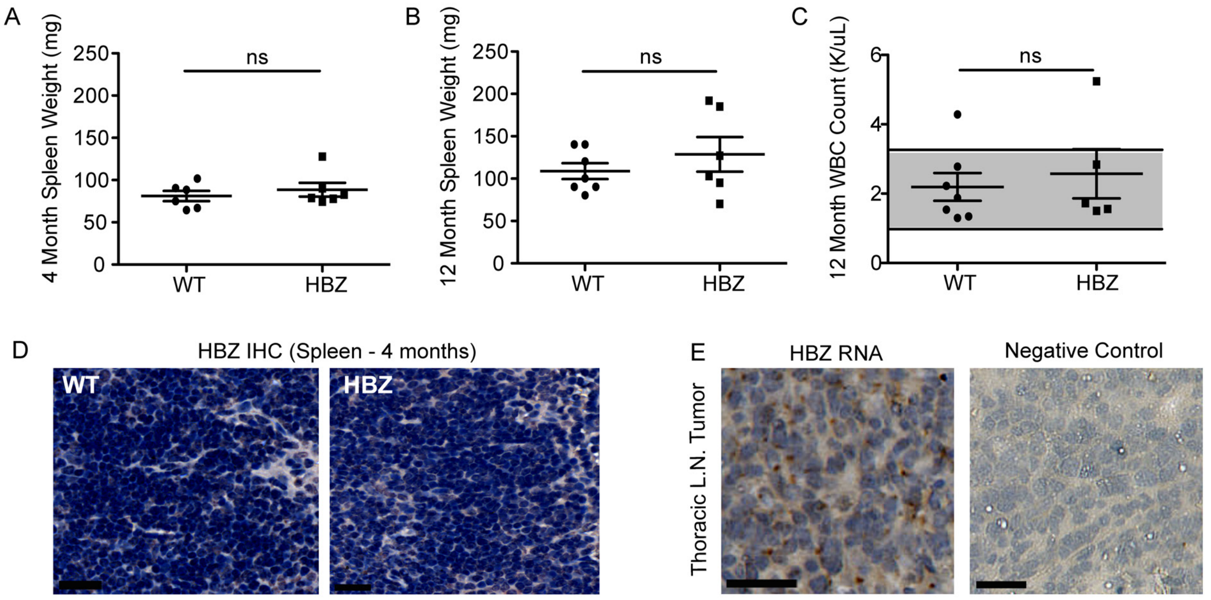

Supplementary Figure 1: Prior to 18 months of age, Gzmb-HBZ mice do not have lymphoproliferative disease and lack HBZ+ cells in spleen. Spleen weight of WT (n=6) and Gzmb-HBZ (n=6) mice at 4 (**A**) and 12 (**B**) months of age. (**C**) WT and Gzmb-HBZ white blood cell (WBC) counts at 12 months. (**D**) Spleen Flag-HBZ IHC in WT and Gzmb-HBZ mice. (**E**) RNA in situ hybridization of HBZ RNA and negative control E. Coli gene *DapB* on HBZ tumor tissue. All data reported as mean +/- SEM. Statistical significance determined by unpaired t-test. $*P \leq 0.05$, $**P \leq 0.01$, $***P \leq 0.001$.

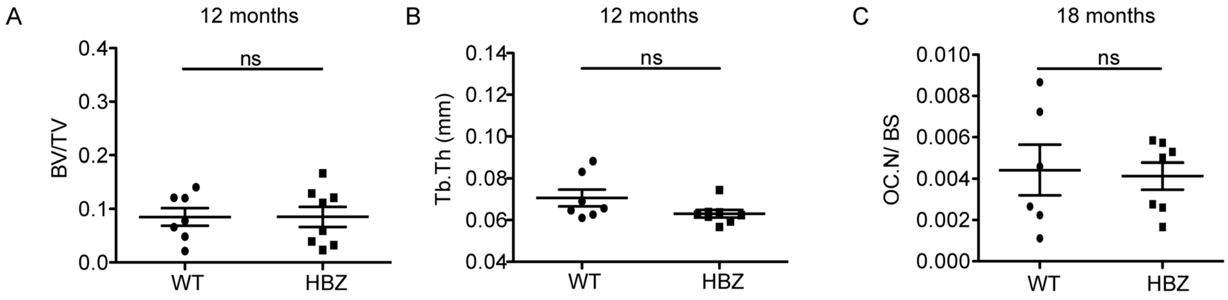

Supplementary Figure 2: 12-month old Gzmb-HBZ mice have normal bone parameters. (**A**) Bone volume/tissue volume (BV/TV) and (**B**) bone trabecular thickness (Tb.Th) of 12- month old WT and Gzmb-HBZ mice (n≥6). (**C**) Osteoclast number/bone surface in WT (n=6) and Gzmb-HBZ (n=7) tibiae at 18 months of age. All data reported as mean +/- SEM. Statistical significance determined by unpaired t-test. $*P \leq 0.05$, $**P \leq 0.01$, $***P \leq 0.001$.
